# Impact of frailty on mortality and healthcare costs and utilization among older adults in South Korea

**DOI:** 10.1038/s41598-023-48403-y

**Published:** 2023-12-01

**Authors:** Fatima Nari, Eun-Cheol Park, Chung- Mo Nam, Sung-In Jang

**Affiliations:** 1 National Cancer Control Institute, National Cancer Center, 323-Ilsan-Ro, Goyang, 10408 Republic of Korea; 2 Institute of Health Services Research, Yonsei University, Seoul, Republic of Korea; 3 Department of Preventive Medicine, Yonsei University College of Medicine, 50 Yonsei-to, Seodaemun-gu, Seoul, 03722 Republic of Korea; 4 Department of Biostatistics, Yonsei University College of Medicine, Seoul, Republic of Korea

**Keywords:** Geriatrics, Health policy, Public health

## Abstract

Frailty has become increasingly relevant in a rapidly aging society, highlighting the need for its accurate identification and exploring associated clinical outcomes. Using a multidimensional framework to estimate frailty in a sample of community dwelling older adults, its effect on mortality, incurred healthcare costs and utilization were investigated. We obtained data from the 2008–2018 Korean Longitudinal Study of Aging (KLoSA). After excluding individuals aged < 65 years and those with missing data, a total of 3578 participants were included in our study. Cox proportional hazard analysis was conducted to investigate the impact of frailty on all-cause mortality by generating hazard ratios (HRs) and population attributable risks (PARs). Healthcare utilization and out-of-pocket costs incurred by frailty were examined using the Generalized Linear Mixed Model (GLMM). Subgroup analyses were conducted according to frailty components. Among 3578 older adults, 1052 individuals died during a 10-year follow up period. Compared to the low risk frailty group, the moderate risk group (HR: 1.52, 95% CI:1.37–1.69) and severe risk group (HR: 3.10, 95% CI: 2.55–3.77) had higher risks for all-cause mortality. 27.4% (95% CI: 19.0–35.3%) of all-cause mortality was attributable to frailty, and the PARs ranged from 0.5 to 22.6% for individual frailty components. Increasing frailty levels incurred higher total healthcare costs and cost per utilization, including inpatient and outpatient costs. Frailty also increased likelihood of inpatient use, longer length of stay and more frequent outpatient visits. Among the frailty components, Basic Activities of Daily Living (BADL) and Instrumental Activities of Daily Living (IADL) in particular were linked to elevated mortality, higher incurred healthcare costs and utilization. Frailty-tailored interventions are of utmost relevance to policy makers and primary caregivers as frailty threatens the ability to maintain independent living and increases risk of detrimental outcomes such as mortality and increased utilization and out-of-pocket costs of healthcare in older adults.

## Introduction

With the extension of average life expectancies worldwide, burden associated with the aging population has been on the rise as well. In Korea, the percentage of older adults over 65 years made up about 16.4% of the total population in 2020, showing an approximately 0.8% average increase from 15.5% and 14.8% in 2019 and 2018, respectively^1^. In a rapidly aging society, where capacity for independent living is becoming increasingly relevant, geriatric syndromes such as frailty have been receiving attention by researchers. While considered to be independent of biological age, frailty is characterised by increased vulnerability to stressors that threatens ability to maintain independence, and puts an individual at greater risk of disability, falls, fractures, hospitalizations, and death^2–4^.

Healthcare utilization and expenses is expected to increase with age, associated with health deterioration, presence of comorbidities, functional limitations and frailty^5^. It has been previously reported that the South Korean older adult demographic accounted for 39.9% of total annual medical expenses in 2017^6^. In extant literature, frailty is considered to be somewhat of a precursor state to resultant disability, while also demonstrating a reversible nature that allows for remission of frailty state^7^.

This knowledge is essential to primary care providers and calls for the recognition and accurate evaluation of frailty in older adults. Numerous models have been proposed to assess frailty, starting from the original Cardiovascular Health Study (CHS) criteria for frailty phenotype^3^. More recently, researchers began to recognise the multidimensional properties of frailty, which led to the development and recommendation of several frailty assessment tools for use in primary care^8^.

Among those, the Multidimensional Prognostic Index (MPI) stood out for showing predictive power and accuracy in predicting negative health outcomes such as mortality, disability, and hospitalization. The MPI is based on items derived from the comprehensive geriatric assessment (CGA) which collects information on physiological, as well as nutritional, functional, and social domains such as comorbidities and cohabitation status^.^^9^. While primarily used to assess risk in a clinical setting, its use has expanded to the community setting as well.

However, little research has investigated the prognostic value of the MPI and longitudinal impact of frailty on detrimental outcomes in community-dwelling older adults in South Korea. Thus, we aimed to explore the influence of multidimensional frailty on mortality, healthcare costs and utilization on older South Korean adults.

## Methods

### Data and study population

The dataset used in our study was the Korean Longitudinal Study of Aging (KloSA) from 2008 to 2018 (2nd to 7th wave). The KloSA is an ongoing, large-scale study conducted biennially by the Korean Labor Institute to collect nationally representative longitudinal data using multistage, stratified sampling from all regions across Korea.

Data on 8,688 participants in 2008 was extracted and followed up over a 10-year period. Exclusion criteria included those under 65 years of age (N = 4651), and with missing information on variables (N = 459), resulting in exclusion of a total of 5110 participants. The final study population consisted of 3578 older adults aged ≥ 65 years.

### Measures

The primary outcomes of this study were all-cause mortality, healthcare costs and healthcare utilization. All-cause mortality was determined from date of enrollment at baseline to time of death, which was confirmed by death certificates data, or censoring over a 10-year maximum follow up period. Healthcare costs were reported as out-of-pocket expenses spent for the past 12 months during all waves from 2008 to 2018 based on the respondent’s recollection. They included (1) inpatient cost—total costs incurred from all hospital admissions due to illnesses in the past 12 months, (2) outpatient cost—total costs incurred from all outpatient physician visits in the past 12 months and (3) total cost—the sum of inpatient and outpatient costs in the past 12 months. Costs were reported in Korean won (1 American dollar = 1101 Korean won; 2008 exchange rate)^10^. Healthcare utilization included (1) inpatient use—the number of hospital admissions due to illnesses in the past 12 months, (2) length of stay—the number of inpatient days in the past 12 months and (3) outpatient use—the number of outpatient physician visits in the past 12 months. Cost per one utilization was also calculated for inpatient cost and outpatient cost. Our study’s main variables and covariates were measured in the 2008 wave.

The study’s main independent variable of interest was frailty measured using the MPI. The MPI was calculated using information from prior literature, albeit with some modifications based on availability of data in our study’s dataset^11,12^. The 8 MPI domains were determined as follows; physical functioning and disability were measured through (1) BADL and (2) IADL, where degree of disability was measured by experiencing any difficulty in performing the seven BADL items (dressing, washing, bathing, eating, moving in and out of bed, going to the toilet, controlling continence) or the ten IADL items (using transportation, going out a short distance, making/receiving phone calls, managing financial matters, doing household chores, meal preparation, shopping, self-care, taking medications, and doing the laundry, (3) physical activity was measured using standard cutoffs of the Korean version of International Physical Activity Questionnaire (IPAQ) to low, moderate, and high activity, with evidence of its validity being reported elsewhere^13^, nutritional status was measured using (4) body mass index, with evidence behind this rationale reported elsewhere^11,14–16^, (5) number of comorbidities were reported; (6) the number of drugs used to treat those comorbidities were reported; (7) cohabitation status was reported; cognitive status was reported using the (8) Korean Mini-Mental State Examination (MMSE)^17^. The operationalization of the MPI variable is fully described in the supplementary material (Supplementary Table S1). A score of 0, 0.5, and 1 were assigned to low, moderate, and severe risk conditions for each domain, respectively. The scores of the eight domains were added and divided by 8 to obtain a score that could range from 0 to 1, with higher values indicating higher risk of frailty. The sum of MPI scores were categorized using previously determined cutoff points from other studies, which were low (< 0.33), moderate (0.33–0.66), and severe (> 0.66) risk of frailty, respectively.

The covariates included in our study consisted of: gender, age (65–69, 70–74, 75–79, 80–84, ≥ 85), region (urban, rural), education (lower than middle school, middle school graduate, high school graduate, university graduate), income (household income divided into quartiles), economic activity (active, inactive), medical coverage type [national health insurance, medical aid (for low-income beneficiaries)], private insurance, smoking, drinking, depression (based on the short form Center for the Epidemiological Studies-Depression 10-item (CESD-10) scale, where presence of depression was determined as having 3 points or more on the scale), participation in social activities (participation in at least one activity such as religious meetings, social gatherings, alumni gatherings, volunteer work). In addition, presence of medical conditions was assessed by asking participants if they received a physician diagnosis of hypertension, diabetes, cancer, liver disease, lung disease, cardiovascular disease, cerebrovascular disease, psychiatric disease, and rheumatism/arthritis.

### Statistical analysis

Our study participants’ general characteristics were reported as frequencies and percentages for categorical variables and mean and standard deviation values for continuous variables. Differences between survivors and those who died during the follow up period of the study were evaluated with the chi-square test. T-tests and analysis of variance (ANOVA) were conducted to examine the distribution of the general characteristics of the study population according to healthcare expenditure and utilization.

The impact of frailty on mortality was determined by adjusted hazard ratios (HR) and 95% confidence intervals (CI) using Cox proportional hazard model. Population attributable risk (PAR) was used to express how much of the mortality can be attributed to certain risk factors or exposures i.e. frailty. Subgroup analyses for the HRs and PARs of frailty and its individual components on all-cause mortality were performed. The PARs of frailty and its components were calculated using the following equation^18,19^.

$$\mathrm{PAR}=\{Pd(\mathrm{HR}-1)/(Pd(\mathrm{HR}-1)+1)\}\times 100$$

where Pd is the proportion of population that has the condition, and HR is the adjusted hazard ratio obtained from the Cox regression model that is associated with the condition. 95% confidence intervals (CI) for the PARs were calculated as well.

Generalized linear mixed models (GLMM) were conducted with link identity function to calculate the association between frailty and healthcare cost and utilization. GLMM is a flexible model, considered to be an extension of generalized linear models with added random effects and can be employed for handling missingness in longitudinal data, in addition to skewed healthcare cost data^20,21^. For all analyses, P values of < 0.05 was considered statistically significant. All statistical analyses were conducted using SAS software (version 9.4).

### Ethical consideration

Our study was reviewed and approved by the International Review Board of Yonsei University’s Health System (IRB number: 4-2021-0112) and adheres to the tenets of the Declaration of Helsinki. Our study did not need to address any ethical concerns because the KLoSA is a publicly available anonymized dataset without any individual identifying information.

## Results

Table 1 shows the general characteristics and distribution of 3578 older adults, stratified by survival status. Over a follow up period of 10 years, 1052 individuals (29.4%) of the total study population were confirmed to have died. When categorized by frailty risk and survival status, 63.4% of participants in the severe risk group, 38.3% of the participants in the moderate risk group, and 22.0% in the low risk group died.

**Table 1:** General characteristics of baseline study population by survival status (2008–2018).

Variables	Total	All-cause mortality		P-value
		Yes	No	
	N (%)	N (%)	N (%)	
Total	3578 (100.0)	1052 (29.4)	2526 (70.6)	
Frailty				< .0001
Low risk	2076 (58.0)	456 (22.0)	1620 (78.0)	
Moderate risk	1420 (39.7)	544 (38.3)	876 (61.7)	
Severe risk	82 (2.3)	52 (63.4)	30 (36.6)	
Gender				< .0001
Male	1508 (42.1)	532 (35.3)	976 (64.7)	
Female	2070 (57.9)	520 (25.1)	1550 (74.9)	
Age				< .0001
65–69	1198 (33.5)	163 (13.6)	1035 (86.4)	
70–74	1077 (30.1)	269 (25.0)	808 (75.0)	
75–79	685 (19.1)	255 (37.2)	430 (62.8)	
80–84	394 (11.0)	198 (50.3)	196 (49.7)	
85 ≤	224 (6.3)	167 (74.6)	57 (25.4)	
Region				0.2490
Urban	2471 (69.1)	712 (28.8)	1759 (71.2)	
Rural	1107 (30.9)	340 (30.7)	767 (69.3)	
Education level				0.0006
Lower than middle school	2526 (70.6)	794 (31.4)	1732 (68.6)	
Middle school graduate	392 (11.0)	94 (24.0)	298 (76.0)	
High School graduate	456 (12.7)	111 (24.3)	345 (75.7)	
University graduate	204 (5.7)	53 (26.0)	151 (74.0)	
Income level				0.8881
Low	2054 (57.4)	622 (30.3)	1432 (69.7)	
Middle low	722 (20.2)	182 (25.2)	540 (74.8)	
Middle high	505 (14.1)	151 (29.9)	354 (70.1)	
High	297 (8.3)	97 (32.7)	200 (67.3)	
Economic activity				< .0001
Active	768 (21.5)	154 (20.1)	614 (79.9)	
Inactive	2810 (78.5)	898 (32.0)	1912 (68.0)	
Medical coverage type				0.0019
National Health Insurance	3370 (94.2)	971 (28.8)	2399 (71.2)	
Medical aid	208 (5.8)	81 (38.9)	127 (61.1)	
Private insurance				< .0001
No	3269 (91.4)	1006 (30.8)	2263 (69.2)	
Yes	309 (8.6)	46 (14.9)	263 (85.1)	
Smoking				< .0001
Current	509 (14.2)	204 (40.1)	305 (59.9)	
Past	498 (13.9)	181 (36.3)	317 (63.7)	
Never	2571 (71.9)	667 (25.9)	1904 (74.1)	
Drinking				0.2411
Current	955 (26.7)	271 (28.4)	684 (71.6)	
Past	451 (12.6)	180 (39.9)	271 (60.1)	
Never	2172 (60.7)	601 (27.7)	1571 (72.3)	
Depression				< .0001
No	2493 (69.7)	650 (26.1)	1843 (73.9)	
Yes	1085 (30.3)	402 (37.1)	683 (62.9)	
Participation in social activities				< .0001
No	1165 (32.6)	462 (39.7)	703 (60.3)	
Yes	2413 (67.4)	590 (24.5)	1823 (75.5)	
Hypertension				0.2049
No	1997 (55.8)	570 (28.5)	1427 (71.5)	
Yes	1581 (44.2)	482 (30.5)	1099 (69.5)	
Diabetes				0.0020
No	2933 (82.0)	830 (28.3)	2103 (71.7)	
Yes	645 (18.0)	222 (34.4)	423 (65.6)	
Cancer				< .0001
No	3457 (96.6)	997 (28.8)	2460 (71.2)	
Yes	121 (3.4)	55 (45.5)	66 (54.5)	
Lung disease				0.0006
No	3436 (96.0)	992 (28.9)	2444 (71.1)	
Yes	142 (4.0)	60 (42.3)	82 (57.7)	
Liver disease				0.0164
No	3507 (98.0)	1022 (29.1)	2485 (70.9)	
Yes	71 (2.0)	30 (42.3)	41 (57.7)	
Cardiovascular disease				0.1953
No	3265 (91.3)	950 (29.1)	2315 (70.9)	
Yes	313 (8.7)	102 (32.6)	211 (67.4)	
Cerebrovascular disease				< .0001
No	3388 (94.7)	958 (28.3)	2430 (71.7)	
Yes	190 (5.3)	94 (49.5)	96 (50.5)	
Psychiatric disease				0.1432
No	3481 (97.3)	1017 (29.2)	2464 (70.8)	
Yes	97 (2.7)	35 (36.1)	62 (63.9)	
Arthritis/rheumatism				0.0045
No	2505 (70.0)	772 (30.8)	1733 (69.2)	
Yes	1073 (30.0)	280 (26.1)	793 (73.9)	

Table 2 shows the general characteristics of the study population’s healthcare costs and utilization at the 2008 baseline. The mean total cost incurred by the frailty groups were ₩363,333, ₩522,169 and ₩1,394,756 in the low risk, moderate risk, and severe risk group, respectively. The mean number of inpatient use of frailty groups were 0.13 times, 0.24 times, 0.73 times for low risk, moderate risk, and severe risk group, respectively. The mean length of stay was 1.95 days, 4.48 days, and 22.48 days in the low risk, moderate risk, and severe risk group, respectively. For outpatient use, the mean number of visits was 9.34 times, 15.22 times, 16.00 times in the low risk, moderate risk, and severe risk group, respectively.

**Table 2:** General characteristics of the study population's healthcare costs and utilization categorized by frailty status at the 2008 baseline.

Variables	Frailty			
	Low risk	Moderate risk	Severe risk	P-value
	Mean ± SD	Mean ± SD	Mean ± SD	
Total (N = 3578)	N = 2076	N = 1420	N = 82	
Cost (in ₩)				
Total cost	363,333 ± 1,156,606	522,169 ± 1,454,733	1,394,756 ± 2,665,075	0.0003
Inpatient cost	236,373 ± 1,047,835	376,965 ± 1,385,201	1,095,366 ± 2,527,157	0.0004
Outpatient cost	126,961 ± 462,566	145,204 ± 370,356	299,390 ± 821,867	0.2262
Utilization				
Inpatient use	0.13 ± 0.5	0.24 ± 0.7	0.73 ± 1.3	< .0001
Length of stay	1.95 ± 8.6	4.48 ± 17.6	22.48 ± 50.8	< .0001
Outpatient use	9.34 ± 15.3	15.22 ± 31.7	16.00 ± 31.0	0.1476
Cost per utilization (in ₩)				
Inpatient use	201,504 ± 905,747	292,266 ± 1,111,316	690,203 ± 1,252,232	0.0426
Outpatient use	12,465 ± 42,017	14,640 ± 139,272	35,826 ± 177,466	0.4202

Table 3 shows the Cox proportional hazard model results for frailty and all-cause mortality. Compared to the low risk group, moderate risk group (HR) (HR: 1.52, 95% CI:1.37–1.69) and severe risk group (HR: 3.10, 95% CI: 2.55–3.77) had a higher hazard ratio of all-cause mortality.

**Table 3:** Cox proportional hazards model for frailty and all-cause mortality.

Variables	All-cause mortality	
	Adjusted HR	95% CI
Frailty		
Low risk	1.00	–
Moderate risk	1.52	(1.37–1.69)
Severe risk	3.10	(2.55–3.77)
Gender		
Male	1.59	(1.40–1.81)
Female	1.00	–
Age		
65–69	1.00	–
70–74	1.74	(1.47–2.06)
75–79	2.87	(2.42–3.40)
80–84	4.34	(3.64–5.18)
85 ≤	8.75	(7.27–10.53)
Region		
Urban	1.00	–
Rural	0.96	(0.88–1.06)
Education level		
Lower than middle school	1.09	(0.89–1.34)
Middle school graduate	0.96	(0.76–1.22)
High school graduate	0.86	(0.68–1.08)
University graduate	1.00	–
Income level		
Low	0.99	(0.85–1.15)
Middle low	0.88	(0.74–1.05)
Middle high	1.00	(0.84–1.20)
High	1.00	–
Economic activity		
Active	1.00	–
Inactive	1.19	(1.05–1.36)
Medical coverage type		
National health insurance	0.99	(0.84–1.16)
Medical aid	1.00	–
Private insurance		
No	1.00	–
Yes	0.79	(0.63–1.00)
Smoking		
Current	1.79	(1.57–2.04)
Past	1.41	(1.23–1.60)
Never	1.00	–
Drinking		
Current	0.86	(0.76–0.97)
Past	0.98	(0.86–1.11)
Never	1.00	–
Depression		
No	1.00	–
Yes	1.05	(0.96–1.16)
Participation in social activities		
No	1.00	–
Yes	0.79	(0.72–0.86)
Hypertension		
No	1.00	–
Yes	0.93	(0.85–1.02)
Diabetes		
No	1.00	–
Yes	1.29	(1.17–1.43)
Cancer		
No	1.00	–
Yes	1.66	(1.41–1.95)
Lung disease		
No	1.00	–
Yes	1.41	(1.18–1.69)
Liver disease		
No	1.00	–
Yes	1.98	(1.57–2.49)
Cardiovascular disease		
No	1.00	–
Yes	0.98	(0.85–1.12)
Cerebrovascular disease		
No	1.00	–
Yes	1.30	(1.12–1.51)
Psychiatric disease		
No	1.00	–
Yes	1.19	(0.97–1.47)
Arthritis/rheumatism		
No	1.00	–
Yes	0.84	(0.77–0.93)

Table 4 shows the results of generalized linear mixed models (GLMM) for the influence of frailty on healthcare costs and utilization in terms of cost and frequency. Total cost (estimate: ₩145,487, p-value: 0.0002; estimate: ₩1,151,781 p-value: < 0.0001) including inpatient cost (estimate: ₩141,135, p-value: 0.0002; estimate: ₩1,118,227, p-value: < 0.0001) and outpatient cost (estimate: ₩3,584, p-value: 0.7193; estimate: ₩31,926, p-value: 0.1935) of moderate and severe frailty risk group, respectively, were higher compared to the low risk group.

**Table 4:** Results of GLMM for frailty on healthcare costs and utilization.

Variables	Frailty						
	Low risk	Moderate risk			Severe risk		
	β	β	SE	P-value	β	SE	P-value
Cost (in ₩)							
Total cost	Ref	145,487	39,320	0.0002	1,151,781	96,762	< .0001
Inpatient cost	Ref	141,135	37,402	0.0002	1,118,227	92,207	< .0001
Outpatient cost	Ref	3584	9973	0.7193	31,926	24,549	0.1935
Utilization							
Inpatient use	Ref	0.066	0.011	< .0001	0.279	0.027	< .0001
Length of stay	Ref	3.312	0.614	< .0001	31.952	1.496	< .0001
Outpatient use	Ref	1.479	0.446	0.001	−0.536	1.080	0.620
Cost per utilization (in ₩)							
Inpatient use	Ref	99,682	31,234	0.0014	732,153	77,276	< .0001
Outpatient use	Ref	200	1255	0.8734	6137	3043	0.0437

*Adjusted for all covariates.

Similarly, frequency of inpatient use (estimate: 0.066, p-value: < 0.0001; estimate: 0.279, p-value: < 0.0001) and length of stay (estimate: 3.312, p-value: < 0.0001; estimate: 31.952, p-value: < 0.0001), of moderate and severe frailty risk group, respectively, were higher compared to the low risk group. Outpatient use of the moderate risk (estimate: 1.479, p-value: 0.001) was significantly higher than that of the low risk group, but that of the severe risk group was not (estimate: −0.536, p-value: 0.620).

Table 5 shows the results of analyses for influence of frailty components on all-cause mortality while adjusting for confounders. Compared to low risk, moderate and high risk of MMSE (HR: 1.18, 95% CI: 1.03–1.35; HR: 1.53, 95% CI: 1.36–1.72), BADL (HR: 2.25, 95% CI: 1.73–2.91; HR: 2.82, 95% CI: 2.37–3.35), IADL (HR: 1.50, 95% CI: 1.26–1.79; HR: 2.26, 95% CI: 2.00–2.55), and physical activity (HR: 1.59, 95% CI: 1.26–2.01; HR: 1.56, 95% CI: 1.38–1.76), had increased risk of mortality, respectively. Those at high risk by BMI (HR: 1.36, 95% CI: 1.22–1.50) and cohabitation status (HR: 1.26, 95% CI: 1.10–1.44), had also a significant higher risk, and also those at moderate risk by number of comorbidities (HR: 1.29, 95% CI: 1.09–1.53).

**Table 5:** Subgroup analyses of frailty components and all-cause mortality.

Variables	All-cause mortality	
	Adjusted HR	95% CI
MMSE		
Low risk	1.00	–
Moderate risk	1.18	(1.03–1.35)
High risk	1.53	(1.36–1.72)
BADL		
Low risk	1.00	–
Moderate risk	2.25	(1.73–2.91)
High risk	2.82	(2.37–3.35)
IADL		
Low risk	1.00	–
Moderate risk	1.50	(1.26–1.79)
High risk	2.26	(2.00–2.55)
Physical activity		
Low risk	1.00	–
Moderate risk	1.59	(1.26–2.01)
High risk	1.56	(1.38–1.76)
BMI		
Low risk	1.00	–
Moderate risk	0.93	(0.83–1.04)
High risk	1.36	(1.22–1.50)
Cohabitation status		
Low risk	1.00	–
Moderate risk	1.08	(0.95–1.23)
High risk	1.26	(1.10–1.44)
Number of medications		
Low risk	1.13	(0.81–1.56)
Moderate risk	1.14	(1.00–1.30)
High risk	1.07	(0.77–1.50)
Number of comorbidities		
Low risk	1.00	–
Moderate risk	1.29	(1.09–1.53)
High risk	1.42	(0.99–2.03)

*Adjusted for all covariates.

Table 6 shows the subgroup analyses results of PARs for all-cause mortality by risk of frailty and its individual components. For both frailty and its individual domains, the PARs were calculated by combining the high risk and moderate risk group into one group and comparing it to the low risk group. The highest PAR could be attributed to the overall frailty which was 27.4% (18.9–35.5%). The PARs of the individual frailty components ranged from 0.5% (−4.9 to 5.8%) for higher risk of number of medications to 22.6% (13.8–31.0%) for higher risk of physical activity.

**Table 6:** Population Attributable Risk (PAR%) according to Frailty Components.

Variables	Population attributable risk	
	PAR (%)	95% CI (%)
Frailty	27.4	(18.9 to 35.5)
MMSE	17.6	(8.7 to 26.2)
BADL	4.3	(0.4 to 8.1)
IADL	10.7	(3.6 to 18)
Physical activity	22.6	(13.8 to 31.0)
BMI	6.8	(3.5 to 10.1)
Cohabitation status	12.8	(4.0 to 21.5)
Number of medications	0.5	(−4.9 to −5.8)
Number of comorbidities	8.9	(−2.0 to −19.6)

We compared general characteristics of the baseline population to the missing population with and without missing frailty values, respectively, in the supplementary material (Supplementary Table S2). The results of the subgroup analyses of frailty components on healthcare costs, healthcare utilization and cost per utilization can be found in the supplementary material (Supplementary Tables S3, S4, S5). In order to differentiate between out-of-pocket healthcare costs due to chronic diseases and frailty separately, we also presented results for patients with frailty including chronic disease, as well patients with frailty but no chronic disease in the supplementary material (Supplementary Table S6).

## Discussion

Our study’s main results indicated that older adults with higher levels of frailty were more likely to die, were hospitalized more frequently and for longer duration, and spent more out-of-pocket expenses on healthcare. Comparing our study’s results proved difficult due to absence of consensus on frailty identification tools and classifications. Furthermore, the conceptualization of the MPI differs from the original definition of frailty phenotype, as it takes into account aspects such as social participation and disability, in addition to physical frailty. It should be noted that while physical frailty is considered to be a precursor to disability, MPI captures a more advanced stage of frailty progression through BADL and IADL, making it less susceptible to remission and tailored interventions. Nevertheless, a meta-analyses showed a dose–response relationship between higher degree of frailty and higher risk of future hospitalization^22^. A study conducted in the Swedish general population revealed that the MPI was predictive of more hospitalization days and shorter survival time over a period of 10–12 years^23^.

On the other hand, while moderate frailty was associated with more frequent outpatient visits, the trend was not linear and showed a lower estimate of outpatient use in the severe frailty group. A prior study reported that higher level of frailty measured by accumulation of deficits was not significantly associated with outpatient emergency department visits^24^. Similarly, another study also reported that while frailty was associated with higher risk of hospitalizations and emergency department visits, they were not associated with outpatient specialist visits. The authors attempted to explain this paradox by suggesting the possibility that due to the nonacute presentation of frailty symptoms, patients found little expected benefits and lack of follow up by physicians leading to underutilization of these services^25^. In addition, we hypothesized that those with severe risk of frailty were more likely to have life threatening illnesses which leads to immediate hospitalization and emergency department visits rather than outpatient use.

Among the eight MPI components, BADL and IADL seemed to generally show a trend of having increased risk of all-cause mortality, higher incurred healthcare costs and use. This was supported by other studies that named BADL and IADL limitation in older adults as the main drivers for mortality, hospitalizations and increased healthcare expenses^26^.

Our subgroup analysis results indicated that the PAR of the MPI measured frailty was higher compared to those of the individual components. Our results also showed that after overall frailty, the component with the highest PAR was physical activity in our study population. This is in contrast with HR estimate results where BADL and IADL showed the highest risk of our study outcomes among all MPI components. However, since the PAR captures both the strength of the association and prevalence of the exposure in the population, factors with low prevalence such as BADL and IADL may present deceptively low PAR values. For this reason, these results must be interpreted with caution. Interestingly, our findings also found a negative non-significant relationship between increasing number of comorbidities and increased healthcare costs and use. This could be due to lack of provision of proper healthcare resources to older adults with multiple comorbidities. Furthermore, Kim et al. reported in their study that older Korean adults had about an average of 2.4 comorbidities, and that only certain diseases such as hypertension, diabetes, and arthritis were linked to frailty^27^.

In addition, when we looked at out-of-pocket healthcare costs incurred by frailty with and without comorbidities separately, patients with both comorbidities and frailty had significantly higher healthcare costs when compared to patients with frailty but no comorbidities. This could be because most chronic diseases have definite diagnostic and treatment criteria leading to increased healthcare expenses, while there is limited knowledge regarding frailty treatment^28^. Despite this, non-comorbidity frailty incurred costs are no less significant and should not be overlooked, particularly in the severe risk group, and this finding has been reported by prior literature as well^5^.

Our study has some strengths. One of them included the use of the nationally representative dataset of the Korea Longitudinal Study of Aging (KloSA). The national representativeness was ensured by the use of multi-stage stratified sampling from all regions around Korea with the exception of Jeju Island. Additionally, GLMM was employed in order to handle unbalanced data with correlated outcomes and missing data. The outcomes in question were healthcare costs and utilization. Employing GLMM reduces bias as it is insensitive to missingness which is common in longitudinal panel data^29^. Furthermore, since the number of missing (mostly due to frailty) in this study was not a negligible amount, we carried out a comparison of general characteristics between individuals with and without missing frailty values. As our results did not show any noticeable differences between the aforementioned populations, we could rule out selection bias in relation to these variables. Also, to our knowledge, this study is among the first few to attempt to observe the association between a CGA-based model of frailty and adverse health outcomes such as mortality, healthcare costs and utilization over a prolonged period of time in South Korean older adults.

Nevertheless, this study is not without its share of limitations. First, due to the difference in conceptualizations and definitions of frailty in existing literature, it was difficult to compare our study’s result with those of other results. However, this limitation is not only applicable to our study, but to other frailty studies as well. Second, due to data limitations, other potential healthcare utilization variables, such as institutionalization. and cost-related variables could not be considered in our study. Institutionalization is one of the associated outcomes of frailty, and a previous study conducted in Korea revealed that frailty was linked to healthcare settings transitions from home to institutions in pneumonia patients^30^. In addition, only out-of-pocket healthcare costs were available in this study, thus we could not include important cost variables such as global health care costs. Third, a lot of variables in our study were based on self-reported measures which may lead to recall bias or over- or under- estimation of data.

## Conclusion

Based on our findings, we concluded that frailty in community-dwelling older adults was associated with elevated mortality risk, more frequent utilization of health services and higher incurred out-of-pocket healthcare costs.

Overall, accurate evaluation and prevention of frailty in the community is essential not only to improve health outcomes of older adults, but also reduce unnecessary and excessive utilization of health services and inflation of healthcare costs.

From a clinical and health policy perspective, recognition and accurate assessment of frailty is essential because it may assist in resource‐planning and shaping health and social policies and interventions aimed at reducing frailty and subsequent mortality and out-of-pocket healthcare costs in the South Korean population.

### Supplementary Information

Supplementary Tables.

## Data Availability

The data used in this study is available at https://survey.keis.or.kr/eng/klosa/databoard/List.jsp.
