# Supplementary material for: Impact of frailty on mortality and healthcare costs and utilization among older adults in South Korea
Source: Sci Rep. 2023 Dec 1;13:21203. doi: 10.1038/s41598-023-48403-y (PMC10692079; doi:10.1038/s41598-023-48403-y)
Supplement: Supplementary file 1 — Supplementary Tables. [file 41598_2023_48403_MOESM1_ESM.docx]

| **Supplementary Table S1. Operationalization of Frailty using the MPI** | | | |
| --- | --- | --- | --- |
| **Tool** | **Low risk =0** | **Moderate risk = 0.5** | **High risk = 1** |
| **MMSE** | ≥27 | 24-26 | ≤ 23 |
| **BADL** | 0-2 | 3-4 | >4 |
| **IADL** | 0-2 | 3-4 | >4 |
| **Physical activity** | High | Moderate | Low |
| **BMI** | 20-25 | ≥ 25 | <20 |
| **Cohabitation status** | Living with others | Living only with spouse | Living alone |
| **Number of medications** | 0-1 | 2-3 | ≥ 4 |
| **Number of comorbidities** | 0-1 | 2-3 | ≥ 4 |

|  | **Supplementary Table S2. General Characteristics of Baseline Study Population and Missing Population by Frailty** | | | | | | |
| --- | --- | --- | --- | --- | --- | --- | --- |
| **Variables** | | **Frailty** | | | | |  |
|  |  | **Study Population** | | | **Missing Population** | |  |
|  |  | **N or**  **mean** | **(% or SD)** | **N or mean** | | **(% or SD)** |  |
| **Total** | | **3,634** | **(100)** | **403** | | **(100)** |  |
| **Gender** | |  |  |  | |  |  |
| Male | | 1,521 | (41.9) | 156 | | (38.7) |  |
| Female | | 2,113 | (58.1) | 247 | | (61.3) |  |
| **Age (in years)** | | 73.6 | (6.3) | 76.2 | | (7.7) |  |
| **Region** | |  |  |  | |  |  |
| Urban | | 2,520 | (69.3) | 285 | | (70.7) |  |
| Rural | | 1,114 | (30.7) | 118 | | (29.3) |  |
| **Education level** | |  |  |  | |  |  |
| Lower than middle  school | | 2,579 | (71.0) | 312 | | (77.4) |  |
| Middle school  graduate | | 399 | (11.0) | 27 | | (6.7) |  |
| High school graduate | | 453 | (12.5) | 43 | | (10.7) |  |
| University graduate | | 203 | (5.5) | 18 | | (4.5) |  |
| **Income level (in ₩)** | | 15,821,921 | 20,003,836 | 15,999,176 | | 20,104,140 |  |
| **Economic activity** | |  |  |  | |  |  |
| Active | | 766 | (21.1) | 59 | | (14.6) |  |
| Inactive | | 2,868 | (78.9) | 344 | | (85.4) |  |

| **Supplementary Table S3. Subgroup Analyses of Frailty Components on Healthcare Costs** | | | | | | | | | |
| --- | --- | --- | --- | --- | --- | --- | --- | --- | --- |
|  | **Cost (in ₩)** | | | | | | | | |
| **Variables** | **Total cost** | | | **Inpatient cost** | | | **Outpatient cost** | | |
|  | **ß** | **S.E** | **P-value** | **ß** | **S.E** | **P-value** | **ß** | **S.E** | **P-value** |
| **MMSE** |  |  |  |  |  |  |  |  |  |
| Low risk | **Ref.** |  |  | **Ref.** |  |  | **Ref.** |  |  |
| Moderate risk | -69,328 | 44,787 | 0.1217 | -59,431 | 42,742 | 0.1644 | -8,962 | 11,312 | 0.4283 |
| High risk | -8,819 | 41,613 | 0.8322 | -1,186 | 39,561 | 0.9761 | -6,881 | 10,502 | 0.5123 |
| **ADL** |  |  |  |  |  |  |  |  |  |
| Low risk | **Ref.** |  |  | **Ref.** |  |  | **Ref.** |  |  |
| Moderate risk | 662,550 | 145,737 | <.0001 | 663,462 | 139,316 | <.0001 | 1,279 | 37,235 | 0.9726 |
| High risk | 1,801,317 | 97,663 | <.0001 | 1,736,877 | 93,076 | <.0001 | 60,183 | 24,945 | 0.0159 |
| **IADL** |  |  |  |  |  |  |  |  |  |
| Low risk | **Ref.** |  |  | **Ref.** |  |  | **Ref.** |  |  |
| Moderate risk | 518,141 | 82,150 | <.0001 | 490,531 | 78,436 | <.0001 | 23,727 | 20,898 | 0.2562 |
| High risk | 884,798 | 60,928 | <.0001 | 829,782 | 58,040 | <.0001 | 52,421 | 15,493 | 0.0007 |
| **Physical activity** |  |  |  |  |  |  |  |  |  |
| Low risk | **Ref.** |  |  | **Ref.** |  |  | **Ref.** |  |  |
| Moderate risk | 9,429 | 80,616 | 0.9069 | 50,611 | 77,077 | 0.5114 | -41,718 | 20,372 | 0.0406 |
| High risk | 110,360 | 40,869 | 0.0069 | 126,354 | 38,908 | 0.0012 | -15,544 | 10,319 | 0.132 |
| **BMI** |  |  |  |  |  |  |  |  |  |
| Low risk | **Ref.** |  |  | **Ref.** |  |  | **Ref.** |  |  |
| Moderate risk | 26,215 | 41,195 | 0.5246 | 3,447 | 38,951 | 0.9295 | 24,561 | 10,374 | 0.0179 |
| High risk | 169,006 | 47,367 | 0.0004 | 166,981 | 44,834 | 0.0002 | -903 | 11,933 | 0.9397 |
| **Cohabitation status** |  |  |  |  |  |  |  |  |  |
| Low risk | **Ref.** |  |  | **Ref.** |  |  | **Ref.** |  |  |
| Moderate risk | 15,234 | 47,663 | 0.7493 | 14,267 | 45,237 | 0.7525 | -866 | 12,022 | 0.9425 |
| High risk | -27,019 | 52,275 | 0.6053 | -15,611 | 49,438 | 0.7522 | -14,031 | 13,174 | 0.2869 |
| **Number of medications** |  |  |  |  |  |  |  |  |  |
| Low risk | **Ref.** |  |  | **Ref.** |  |  | **Ref.** |  |  |
| Moderate risk | 247,770 | 49,602 | <.0001 | 197,708 | 47,268 | <.0001 | 51,579 | 12,526 | <.0001 |
| High risk | 309,244 | 135,709 | 0.0227 | 273,886 | 129,285 | 0.0342 | 48,840 | 34,268 | 0.1541 |
| **Number of comorbidities** |  |  |  |  |  |  |  |  |  |
| Low risk | **Ref.** |  |  | **Ref.** |  |  | **Ref.** |  |  |
| Moderate risk | -124,630 | 68,525 | 0.069 | -115,218 | 64,788 | 0.0754 | -8,132 | 17,267 | 0.6377 |
| High risk | -145,109 | 145,503 | 0.3186 | -93,558 | 137,649 | 0.4967 | -48,406 | 36,670 | 0.1868 |
| **adjusted for other covariates* | | | | | | | | | |

| **Supplementary Table S4. Subgroup Analyses of Frailty Components on Healthcare Utilization** | | | | | | | | | |
| --- | --- | --- | --- | --- | --- | --- | --- | --- | --- |
| **Variables** | **Utilization** | | | | | | | | |
|  | **Inpatient use** | | | **Length of stay** | | | **Outpatient use** | | |
|  | **ß** | **S.E** | **P-value** | **ß** | **S.E** | **P-value** | **ß** | **S.E** | **P-value** |
| **MMSE** |  |  |  |  |  |  |  |  |  |
| Low risk | **Ref.** |  |  | **Ref.** |  |  | **Ref.** |  |  |
| Moderate risk | 0.004 | 0.012 | 0.752 | -0.331 | 0.691 | 0.632 | 1.004 | 0.486 | 0.039 |
| High risk | 0.025 | 0.012 | 0.036 | 2.220 | 0.661 | 0.001 | 1.061 | 0.475 | 0.026 |
| **ADL** |  |  |  |  |  |  |  |  |  |
| Low risk | **Ref.** |  |  | **Ref.** |  |  | **Ref.** |  |  |
| Moderate risk | 0.123 | 0.040 | 0.002 | 11.412 | 2.159 | <.0001 | 1.082 | 1.574 | 0.492 |
| High risk | 0.393 | 0.028 | <.0001 | 54.145 | 1.481 | <.0001 | -3.851 | 1.103 | 0.001 |
| **IADL** |  |  |  |  |  |  |  |  |  |
| Low risk | **Ref.** |  |  | **Ref.** |  |  | **Ref.** |  |  |
| Moderate risk | 0.144 | 0.023 | <.0001 | 3.397 | 1.255 | 0.007 | 1.731 | 0.898 | 0.054 |
| High risk | 0.204 | 0.017 | <.0001 | 21.917 | 0.949 | <.0001 | 2.544 | 0.691 | 0.0002 |
| **Physical activity** |  |  |  |  |  |  |  |  |  |
| Low risk | **Ref.** |  |  | **Ref.** |  |  | **Ref.** |  |  |
| Moderate risk | 0.066 | 0.022 | 0.003 | 0.743 | 1.227 | 0.545 | -1.456 | 0.854 | 0.088 |
| High risk | 0.037 | 0.012 | 0.001 | 2.759 | 0.642 | <.0001 | -0.816 | 0.457 | 0.074 |
| **BMI** |  |  |  |  |  |  |  |  |  |
| Low risk | **Ref.** |  |  | **Ref.** |  |  | **Ref.** |  |  |
| Moderate risk | 0.013 | 0.012 | 0.283 | -1.151 | 0.684 | 0.092 | 1.314 | 0.508 | 0.010 |
| High risk | 0.060 | 0.014 | <.0001 | 3.854 | 0.781 | <.0001 | -0.257 | 0.578 | 0.656 |
| **Cohabitation status** |  |  |  |  |  |  |  |  |  |
| Low risk | **Ref.** |  |  | **Ref.** |  |  | **Ref.** |  |  |
| Moderate risk | 0.011 | 0.014 | 0.432 | 0.360 | 0.768 | 0.640 | 1.002 | 0.558 | 0.073 |
| High risk | -0.001 | 0.016 | 0.931 | -0.007 | 0.869 | 0.994 | 1.249 | 0.649 | 0.054 |
| **Number of medications** |  |  |  |  |  |  |  |  |  |
| Low risk | **Ref.** |  |  | **Ref.** |  |  | **Ref.** |  |  |
| Moderate risk | 0.103 | 0.014 | <.0001 | 1.973 | 0.777 | 0.011 | 4.102 | 0.550 | <.0001 |
| High risk | 0.186 | 0.038 | <.0001 | 0.404 | 2.130 | 0.850 | 3.777 | 1.512 | 0.013 |
| **Number of comorbidities** |  |  |  |  |  |  |  |  |  |
| Low risk | **Ref.** |  |  | **Ref.** |  |  | **Ref.** |  |  |
| Moderate risk | 0.0005 | 0.021 | 0.982 | -0.545 | 1.137 | 0.631 | -0.118 | 0.844 | 0.888 |
| High risk | -0.035 | 0.043 | 0.425 | -1.686 | 2.403 | 0.483 | -4.539 | 1.777 | 0.011 |
| **adjusted for all covariates* | | | | | | | | | |

| **Supplementary Table S5. Subgroup Analyses of Frailty Components on Cost Per Utilization** | | | | | | |
| --- | --- | --- | --- | --- | --- | --- |
| **Variables** | **Cost per Utilization (in ₩)** | | | | | |
|  | **Inpatient cost** | | | **Outpatient cost** | | |
|  | **ß** | **S.E** | **P-value** | **ß** | **S.E** | **P-value** |
| **MMSE** |  |  |  |  |  |  |
| Low risk | **Ref.** |  |  | **Ref.** |  |  |
| Moderate risk | -59,899 | 35,830 | 0.0946 | -57 | 1,316 | 0.9653 |
| High risk | -13,068 | 32,921 | 0.6914 | 1,551 | 1,347 | 0.2496 |
| **ADL** |  |  |  |  |  |  |
| Low risk | **Ref.** |  |  | **Ref.** |  |  |
| Moderate risk | 400,181 | 117,708 | 0.0007 | -1,568 | 4,279 | 0.7141 |
| High risk | 1,213,250 | 78,159 | <.0001 | 4,050 | 3,146 | 0.198 |
| **IADL** |  |  |  |  |  |  |
| Low risk | **Ref.** |  |  | **Ref.** |  |  |
| Moderate risk | 383,779 | 65,897 | <.0001 | 1,017 | 2,451 | 0.6783 |
| High risk | 602,354 | 48,547 | <.0001 | 6,727 | 1,976 | 0.0007 |
| **Physical activity** |  |  |  |  |  |  |
| Low risk | **Ref.** |  |  | **Ref.** |  |  |
| Moderate risk | 46,222 | 64,865 | 0.4761 | -758 | 2,273 | 0.7389 |
| High risk | 94,082 | 32,472 | 0.0038 | 317 | 1,269 | 0.8026 |
| **BMI** |  |  |  |  |  |  |
| Low risk | **Ref.** |  |  | **Ref.** |  |  |
| Moderate risk | 21,424 | 32,077 | 0.5042 | -1,704 | 1,566 | 0.2766 |
| High risk | 128,068 | 37,002 | 0.0005 | 1,231 | 1,777 | 0.4882 |
| **Cohabitation status** |  |  |  |  |  |  |
| Low risk | **Ref.** |  |  | **Ref.** |  |  |
| Moderate risk | 2,662 | 37,521 | 0.9434 | 1,075 | 1,628 | 0.5091 |
| High risk | -18,102 | 40,733 | 0.6568 | 1,791 | 12,094 | <.0001 |
| **Number of medications** |  |  |  |  |  |  |
| Low risk | **Ref.** |  |  | **Ref.** |  |  |
| Moderate risk | 122,018 | 39,502 | 0.002 | 1,747 | 1,522 | 0.251 |
| High risk | 161,712 | 107,982 | 0.1343 | -13,043 | 4,205 | 0.0019 |
| **Number of comorbidities** |  |  |  |  |  |  |
| Low risk | **Ref.** |  |  | **Ref.** |  |  |
| Moderate risk | -66,263 | 53,370 | 0.2144 | -4,994 | 2,599 | 0.0547 |
| High risk | -17,122 | 113,515 | 0.8801 | -8,996 | 5,434 | 0.0979 |
| **adjusted for all covariates* | | | | | | |

| **Supplementary Table S6.**  **Subgroup Analyses of Frailty and Chronic Disease on Healthcare Costs** | | | | | | |
| --- | --- | --- | --- | --- | --- | --- |
|  | **Frailty** | | | | | |
|  | **Comorbidity included** | | | **Comorbidity excluded** | | |
|  | **ß** | **S.E** | **P-value** | **ß** | **S.E** | **P-value** |
| **Total cost (in ₩)** |  |  |  |  |  |  |
| Low risk | **Ref.** |  |  | **Ref.** |  |  |
| Moderate risk | 145,487 | 39,320 | 0.0002 | 31,414 | 35,614 | 0.3778 |
| Severe risk | 1,151,781 | 96,762 | <.0001 | 266,403 | 70,281 | 0.0002 |
| **Inpatient cost (in ₩)** |  |  |  |  |  |  |
| Low risk | **Ref.** |  |  | **Ref.** |  |  |
| Moderate risk | 141,135 | 37,402 | 0.0002 | 29,693 | 33,930 | 0.3816 |
| Severe risk | 1,118,227 | 92,207 | <.0001 | 244,357 | 66,860 | 0.0003 |
| **Outpatient cost (in ₩)** |  |  |  |  |  |  |
| Low risk | **Ref.** |  |  | **Ref.** |  |  |
| Moderate risk | 3,584 | 9,973 | 0.7193 | 630 | 8,197 | 0.9388 |
| Severe risk | 31,926 | 24,549 | 0.1935 | 19,489 | 16,207 | 0.2293 |

**adjusted for all covariates*
